# Safety and efficacy trial of adipose-tissue derived oral preparation V-6 Immunitor (V-6): results of open-label, two-month, follow-up study

**DOI:** 10.1186/1476-511X-9-14

**Published:** 2010-02-02

**Authors:** Aldar S Bourinbaiar, Vichai Jirathitikal

**Affiliations:** 1 Immunitor USA Inc., College Park, MD 20740, USA

## Abstract

**Background:**

Chronic inflammations, atherosclerosis and obesity, are major risk factors for cardiovascular diseases. Immune modulation of the inflammatory response has shown promise in animal models of atherogenesis and metabolic disease. Tableted dietary supplement, V-6, containing pooled antigens derived from pig adipose tissue has been administered daily to 12 volunteers for 2 months.

**Results:**

No significant changes were observed in liver ALT and AST enzymes, i.e., 28 vs 23.8 IU and 22.6 vs 24.8 IU, with p = 0.07 and p = 0.49, respectively. Creatinine decreased; 0.88 vs 0.84 mg/dL (p = 0.05) while BUN moved upward; 14.5 vs 17.5 mg/dL (p = 0.01), but both values remained within normal range. Blood glucose remained within normal range; 96.1 vs 101.1 mg/dL (p = 0.04). Complete blood cell analysis has not revealed any change except slight increase in hemoglobin; 13.13 to 13.96 g/dL (p = 0.0002); hematocrit and red blood cells count 40.3 to 42.3% (p = 0.02) and 5.15 to 5.35 × 10^6 ^cells/mm^3 ^(p = 0.03) respectively. Blood pressure systolic and diastolic values were not affected, i.e., 116.1 vs 116.3 (p = 0.12) and 76.8 vs 76.6 (p = 0.99). Body weight and body mass index (BMI) remained same; 66.4 vs 66.3 kg (p = 0.47) and 25.7 vs 25.6 kg/m^2 ^(p = 0.2). Body fat deposit indices, such as abdomen; mid-arm; and thigh circumferences declined by 3.5 cm (p = 0.008); 1.2 cm (p = 0.004); and 3.0 cm (p = 0.0007) respectively. The total cholesterol and LDL levels did not change; 195.5 vs 195.1 (-0.2%; p = 0.8) and 113.4 vs 120.3 (6.1%; p = 0.08) respectively. Triglycerides have been reduced but not statistically significant; 168.1 vs 118 mg/dL (-29.8%; p = 0.2). In contrast, HDL content had risen by 29.7% from 39.4 to 51.1 mg/dL in all 12 patients (p = 0.000003). TG/HDL ratio - a marker of insulin resistance - was reduced from 4.78 to 2.56 (-46.5%; p = 0.04).

**Conclusions:**

These results demonstrate that V-6 is safe and has a potential as an anti-atherogenic and overweight/obesity immune intervention.

## Background

Coronary heart disease (CHD) is a leading cause of death in industrialized countries [1]. Atherosclerosis and obesity are two principal pathological conditions that predispose to cardiovascular disease (CVD) [2]. The term atherosclerosis, commonly referred to as a "hardening of the arteries", is associated with the formation of lipid-laden plaques within the wall of large arteries. Excessive body fat accumulation characterizes overweight and obesity - conditions that affect more than 60% of the adult population in the United States. Epidemiological studies have shown that high levels of atherogenic low density lipoproteins (LDL) and triglycerides (TG) along with low levels of high density lipoproteins (HDL) or "good cholesterol" are strongly associated with both atherosclerosis and obesity and consequently the risk for CHD [1,2]. The conventional methods for controlling abnormal lipid metabolism are through reduction of dietary intake of fats and treatment with cholesterol and obesity-reducing drugs. The most important class of drugs that influences hypercholesterolemia are statins, which mainly lower the LDL cholesterol. Nicotinic acid and fibrates can induce higher HDL levels but may not be taken regularly because of their side effects. The extent of beneficiary effect of diet is limited and reduction of cholesterol by drugs is often associated with unwanted side effects. Similarly, the effect of obesity drugs has been modest and the attrition rate is an issue that remains to be solved [3]. Thus, alternative means to prevent and/or treat atherosclerotic and metabolic disease have to be found to satisfy the unmet needs.

It is now generally acknowledged that atherosclerosis is an inflammatory disease - an idea that was first advanced by Rudolf Wirchow in 1856 [4]. Recent studies have brought forward the notion that obesity is a chronic inflammation caused by self-directed immune reaction against adipose tissue [5,6]. Modulation of the inflammatory response may represent a valuable strategy to prevent and/or treat both atherosclerosis and obesity [7-10]. The earliest credible attempt of immune intervention has been reported in 1959 by Hungarian scientists Gero et al., who immunized rabbits with lipoproteins isolated from the serum of cockerels [11]. In 1970's Soviet researchers proposed that atherosclerosis is an autoimmune disease and tolerization with low doses of some but not all lipoprotein fractions can prevent atherogenic process [12,13]. Nevertheless, the concept of immune modulation of atherosclerosis has not become fashionable in the West until 1990's. This delay was perhaps due to the skeptical report by Bailey et al., who failed to reproduce the original findings of Gero [14]. Nevertheless, in the past 20 years many experimental approaches, especially vaccines directed against various immunogenic entities involved in lipid metabolism, have demonstrated success in animal models [7-10]. The first human trial of atherosclerosis vaccine was reported in 2003 by Davidson et al. [15]. While their cholesteryl ester transfer protein (CETP) vaccine (CETi-1) was well tolerated and anti-CETP antibodies were induced in patients, no substantial effect on HDL levels has been demonstrated.

If atherosclerosis and obesity are result of self-directed autoimmunity then oral administration of autoantigens may indeed produce the desired immune tolerance, which could counteract the inflammatory process [16]. In this open-label, clinical study, involving 12 individuals, we have evaluated whether oral administration of pooled antigens from adipose tissue is safe and can favorably affect the abnormal lipid metabolism.

## Results

**Figure 1:**
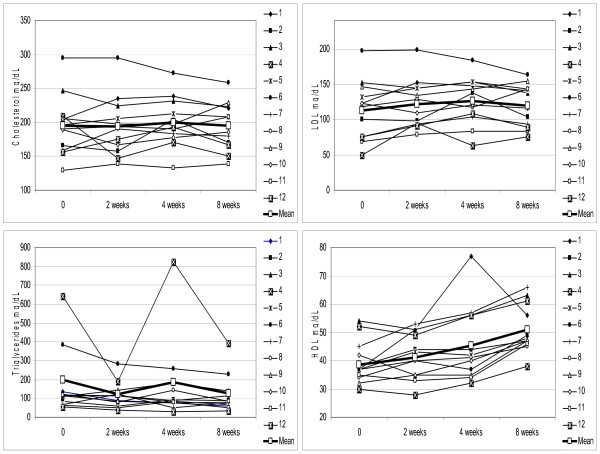
**Changes in total plasma cholesterol (CH; -0.2%; p = 0.76), low density lipoproteins (LDL; +6.1%; p = 0.08), triglycerides (TG; -29.8%; p = 0.24), and high density lipoproteins (HDL; +29.7%; p = 0.000003), resulting from oral administration of V-6 as evaluated by repeated measure ANOVA**. Individual values from each of 12 patients, collected through weeks 2, 4, and 8, are plotted and mean values are shown in each graph in bold.

None of the patients had reported any adverse effect attributed to V-6 treatment, most had noted better mood and quality of life. While subjective, these impressions are corroborated by objective lab analysis results. The serum levels of lipids such as total cholesterol, LDL, HDL and triglycerides have been analyzed at 2 week, 1 month and 2 month intervals after first administered dose of V-6 (Fig. 1). The total cholesterol content has not changed from the baseline value; 195.5 vs 195.1 (p = 0.76). LDL levels fluctuated slightly upward but results were not statistically significant, i.e., 113.4 vs 120.3 (p = 0.08). The cholesterol to LDL ratio has not changed considerably, i.e., 1.86 vs 1.66 (p = 0.17). In contrast, levels of HDL have increased by 29.7% from 39.4 to 51.1 mg/dL (p = 0.000003) in all 12 patients. The average/median increase in HDL at the end of 2 months treatment was equal to 11.7/11 mg/dL (range 5-21 mg/dL; 95% CI 8.7 - 14.6 mg/dL). This change reflected in decrease of cholesterol to HDL ratio by 25% from 5.17 to 3.88 (p = 0.000001). TG levels were reduced in 8 out 12 patients with average intra-group decrease equal to 29.8%, i.e., from 168.1 to 118 mg/dL (p = 0.24). Nevertheless, this change was not statistically significant despite the fact that average TG decrease (-51.9%) among 8 responders has been substantial (-80.9 mg/dL; 95% CI 150.1-11.7) as opposed to modest increase (+6.8%) in non-responders (+11.5 mg/dL; 95% CI 8.4-31.4). This incongruity is likely to be due to high outlier TG values, especially in patient #4, which caused skewed and statistically non-significant results. The removal of patient #4 outlier numbers produced mean 25.4% decrease, i.e., from 124.9 to 93.2 mg/dL and improved the probability value (p = 0.1), but it remained insignificant. The use of repeated measure, non-parametric Friedman test has not produced better significance as obtained p value (0.23) was still above significance level. Paired, two-tailed Student t-test, which compared baseline and end-of-study outcomes of all 12 patients produced p = 0.054, which was still above 0.05 cut-off value. The quasi-linear regression analysis that considers the gap between baseline and end-of-study TG values from outliers (patients #4 and #6) and remaining patients produced p = 0.000000004 with R-squared regression coefficient 0.89. These results indicate that there is a significant trend supporting TG decrease but sample size has been insufficient to make definitive conclusion. The TG/HDL ratio, which is a predictor of insulin resistance and CHD risk, has been reduced by almost half (46.5%; p = 0.036) from mean 4.78 (95% CI 1.11-8.45) to 2.56 (95% CI 0.86-4.26) as evaluated by paired Student t-test.

**Figure 2:**
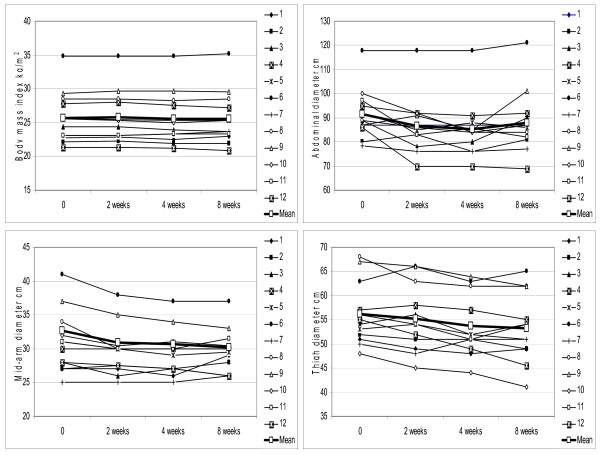
**Negligible effect of daily dose of V-6 on body mass index (BMI; -0.4%; p = 0.21) as opposed to statistically significant reduction in waist (-3.8%; p = 0.008), mid-arm (-3.9%; p = 0.004), and thigh (-5.3%; p = 0.0007) circumferences as followed through weeks 2, 4, and 8**. Individual values from each time-point for every patient are plotted and mean values are shown as bold line.

V-6 effect was measured for changes in body weight and body mass index (BMI). No significant alterations in body weight were found, with average weight prior to and after treatment being 66.37 vs 66.28 kg (p = 0.47). Similar, non-significant decrease was observed with BMI, i.e., 25.7 vs 25.6 kg/m^2 ^(p = 0.21) (Fig. 2). The anthropometric predictors of body fat such as abdomen, mid-arm, and thigh circumferences were evaluated by repeated measure ANOVA (Fig. 2). Waistline decreased in 8 out 12 individuals from average 91.54 to 88.08 cm (3.5 cm; p = 0.008; 95% CI 8.9-2.0 cm). The waist circumference, when stratified to 9 women, declined from abdominal obesity defining level 88.7 cm down to 84.1 cm (4.6 cm; p = 0.001; 95% CI 2.9-12.1 cm). Mid-arm circumference had decreased by 4% in 8 out 12 individuals from average 30.9 cm to 29.7 cm at the end of two months (1.2 cm; p = 0.0035; 95% CI 0.14-2.6 cm). The thigh circumference has been reduced in 10 out of 12 individuals, i.e., 56.17 at baseline vs 53.2 cm (2.96 cm; p = 0.0007; 95% CI 0.8-5.1 cm). The similarity in outcome from all three measured sites of fat deposition indicates that this trend is consistent and statistically significant despite small sample size.

Pre- and post-treatment blood pressure systolic and diastolic values were not affected significantly, i.e., 116.1 vs 116.3 (p = 0.12) and 76.8 vs 76.6 (p = 0.99). No significant changes were observed in liver enzymes profile. ALT and AST levels were not influenced by V-6, i.e., 28 vs 23.8 and 22.6 vs 24.8 with p values 0.07 and 0.49, respectively. Quite contrary, patient #4 who had elevated ALT and AST levels (96 IU and 44 IU) at baseline had experienced liver function improvement (56 IU and 34 IU) at the end of follow-up. V-6 had no adverse effect on kidney function. Creatinine levels appeared to decrease; 0.88 vs 0.84 mg/dL (p = 0.048) while blood urea nitrogen (BUN) has shown a reverse trend; 14.5 vs 17.5 mg/dL (p = 0.014). While statistically significant, both values remained within normal ranges; 0.5-2.0 mg/dL and 9-23 mg/dL for creatinine and BUN, respectively. Blood sugar levels also remained within the normal range (70-130 mg/dL) even though a small upward trend has been observed; 96.1 vs 101.1 (p = 0.04).

Complete blood cell (CBC) analysis has been carried out at regular intervals to identify changes that could be associated with V-6 therapy. Hemoglobin levels increased slightly from 13.13 to 13.96 g/dL (p = 0.0002), which, however, remained within normal range 12.1-17.2 g/dL. This reflected in increase of hemoglobin amount per red blood cell (MCH) from 25.75 up to 26.5 picograms/cell (p = 0.0002), but hemoglobin concentration relative to size of the cell (MCHC) has not changed appreciably, i.e., 32.75 vs 32.92 g/dL (p = 0.18). The average red blood cell size (MCV) increased from 77.5 to 79.8 femtoliters (p = 0.0007). Hematocrit and red blood cells count had increased, but remained within normal range 40.3 to 42.3% (p = 0.015) and 5.15 to 5.35 × 10^6 ^cells/mm^3 ^(p = 0.034) respectively. The number of platelets has moved upward, from 244,333 to 264,166 per mm^3^, but the difference was not significant (p = 0.12). The mean white blood cells (WBC) count has not changed: 7,858 vs 7,633 cells/mm^3 ^(p = 0.65). The percent of leukocytes and neutrophils was not affected by V-6 therapy; 38.5% vs 36.1% (p = 0.78) and 58.5% vs 61.7% (p = 0.44). Although pro-inflammatory eosinophils were seen to decline from mean 4.13% down to 2.33% the significance was not attained (p = 0.29), mainly due to the undetectable levels of such cells at certain time-points in 5 out 12 patients.

## Discussion

The Greek physician Hippocrates observed in 400 BC that "Sudden death is more common in those who are naturally fat than in the lean" [17]. Atherosclerosis and obesity were initially thought as lipid-storage diseases, but are now increasingly recognized as inflammatory conditions, characterized by infiltration of macrophages and T cells, which interact with one another and with atheromas and adipocytes [5,6]. We now know that the connections between obesity and fatty arteries are complicated, but it is clear that inflammation is the underlying cause for these risk factors [1-6]. Our working hypothesis is based on assumption that chronic inflammation is due to self-directed autoimmunity and thus the induction of immune tolerance through oral delivery of autoantigens is a logical approach to overcome both obesity and atherosclerosis.

The seminal work of Gero et al., has laid basis to the ground-breaking concept that modulation of the immune system is a valid strategy to control atherogenic dyslipidemia. While his work was met with initial skepticism, many subsequent studies have confirmed the possibility of inhibiting atherosclerosis by inducing immune response to key antigens involved in lipid metabolism. Gero has used beta-lipoprotein, the main protein in LDL particles, as their anti-atherogenic xenoantigen. Then, Russian and Czech investigators have demonstrated the atheroprotective effect in a series of animal studies by using beta- and pre-beta-lipoproteins, cholesterol, very low density lipoproteins (VLDL), gamma-globulin, albumin, and even Candida albicans, but not LDL [12,13,18,19]. After a period of relative inactivity a sudden surge of interest became apparent in 1990's when several groups in the USA and Western Europe have published the potential of cholesterol, LDL, oxidized form of LDL, beta 2-glycoprotein, heat-shock protein 65 (HSP-65), and avian herpesvirus as vaccine antigens capable of preventing atherosclerosis [20-29]. More recent studies while continuing the investigation of earlier identified antigens [30-35] have focused on additional targets involved in atherogenesis. These included a wide variety of immunogens such as cholesteryl ester transfer protein (CETP), HSP-60, tumor necrosis factor alpha (TNF-α), IL-12, vascular endothelial growth factor receptor 2 (VEGF), angiopoietin-2 receptor (TIE2), CD99, phosphorylcholine, and Streptococcus pneumoniae [36-47]. Recently published studies of obesity vaccines have shown promise with ghrelin and gastric inhibitory polypeptide (GIP) as candidate targets for weight control [8-10]. However, while most animal studies were encouraging, so far, only one vaccine progressed into human trials but was abandoned after phase 2 trial had shown low level (6%) increase in HDL levels [15].

Since there is a lack of adequate immune intervention studies in humans how our data compares with results from cholesterol and obesity drug trials? LDL cholesterol is the main, if not the only, lipid target in the effort to reduce CVD morbidity and mortality [1]. Clinical and epidemiological studies have identified HDL as more promising target independently and inversely associated with an increased risk of CHD [48,49]. LDL-lowering drugs, such as niacin, fibrates, and statins, are not very effective in raising HDL. The meta-analysis of published trials has shown that average HDL elevation in statin trials was 1.6 mg/dL, fibrate trials 2.6 mg/dL, and combinations trials of statins with niacin 12 mg/dL. In terms of percentage, statins, fibrates, and nicotinic acid increase HDL by 5-10%; 10%; and 20% respectively [1]. Our mean 11.7 mg/dL or 29.7% increase in HDL levels observed in all patients compares favorably with best results in the field, i.e., niacin and statin combination. Long term, follow-up studies have demonstrated that incremental HDL elevation either in absolute or percentage figures can predict cardiovascular risk. Goldenberg et al., have shown 29% risk reduction per 5 mg/dL increment in HDL among patients with LDL levels below 130 mg/dL [48]. In other cholesterol-reducing drug trials for every 1% increase in HDL there was a 3% reduction in death or myocardial infarction [49]. If these figures are extrapolated to our findings then risk reduction of CHD due to V-6 intervention is between 68% and 89% - a benefit that surpasses by 2-3 folds the average benefit associated with optimal LDL reduction [1].

The effect of V-6 in reducing triglycerides has been quite substantial but due to power limitation could not be ascertained by every statistical test we have employed. TG/HDL ratio, especially when higher than 3.5, is a strong independent predictor for insulin resistance and cardiovascular mortality [50]. At the end of study the TG/HDL ratio has declined from 4.78 to 2.56 (p = 0.036). This change is accompanied with 25% (p = 0.000001) decrease of cholesterol to HDL ratio - a predictor of atherogenesis and CHD risk. High TG and low HDL is characteristic of patients with the metabolic syndrome, a condition strongly associated with the development of both type 2 diabetes and CHD. V-6 reduced TG/HDL ratio below risk threshold - an observation that supports the potential role of this intervention in management of type 2 diabetes mellitus. Thus, this endpoint needs to be queried further in a larger population of patients.

Currently approved anti-obesity drugs, orlistat, sibutramine, and rimonabant show only limited efficacy and are often associated with unpleasant side-effects, which account for high attrition rate. Orlistat is a gastric lipase inhibitor, sibutramine is a noradrenaline/serotonin reuptake blocker, and rimonabant is an endocannabinoid CB1 receptor antagonist. The meta-analysis of data from obesity drug trials, which included waist circumference as an endpoint, indicates that orlistat therapy reduced WC by 2.06 cm (95% CI 1.3-2.9); sibutramine by 3.99 cm (95% CI 3.3-4.7); and rimonabant by 3.89 cm (95% CI 3.3- 4.5) [3]. Our waistline results are comparable to the outcome from obesity drugs since mean WC reduction was 3.5 cm (95% CI 8.9-2.0 cm). Other anthropometric predictors of body fat, arm and thigh circumference, had declined as well. It also needs to be kept in mind that in obesity drug trials patients were commonly subjected to low-calorie diet, exercise, and behavioral modification in addition to drug intervention. In our group none of the patients had changed their usual diet, quite contrary, all patients, except one (#4), had reported increased appetite and food intake. This perhaps explains why there were no significant changes in body weight and BMI.

A substantial body of evidence exists which indicates that dietary magnesium can influence atherogenesis through reduction in cholesterol, LDL, and TG levels [51-53]. At the same time, a marginal increase in HDL levels (2.5 mg/dL) has been reported [53,54]. The summary of clinical outcomes can be found in the review paper published by Rosanoff and Seelig in which they indicated that magnesium supplements can lower CH, LDL, and TG by 6-23%, 10-18%, and 10-42% respectively and increase HDL by 4-11% [55]. To the best of our knowledge there is no published evidence that magnesium alone can increase HDL levels by ~30% or ~12 mg/dL or reduce abdominal fat in a statistically significant manner. As magnesium is the carrier of adipose-derived antigens in V-6 tablet, one may argue that our results are due to non-specific magnesium supplementation. However, it is unlikely that magnesium alone can augment HDL and decrease fat deposit indices. If this was true, we would expect much greater effect on CH and LDL levels than on HDL. Nevertheless, a placebo study employing the same dose of magnesium as in V-6 tablets needs to be conducted to rule out this possibility.

Our findings indicate that V-6 is safe and despite small sample size had significantly increased HDL levels and reduced obesity indices. None of the patients reported any unpleasant side-effects or feelings. Quite contrary they were highly satisfied with V-6 treatment - these impressions, however, can be dismissed as subjective. Nevertheless, none of the measured safety parameters such as kidney and liver functions, blood pressure, glucose levels, and CBC results have been affected in any appreciable manner. CBC analysis has not revealed any noticeable changes in blood picture except statistically significant increase in hemoglobin, hematocrit and erythrocyte levels albeit within normal range. Since levels of hemoglobin below 13 g/dL are strongly associated with higher risk of coronary artery disease this finding might be interpreted as a beneficial effect resulting from V-6 administration [56].

The magnitude of clinical response to V-6 was comparable to the results obtained in clinical trials of cholesterol and obesity drugs. This is the first observation whereby both cardiovascular risk factors were affected by a single immune intervention. What is the mechanism of V-6 action? Prior atherosclerosis vaccine studies have been quite consistent that small rather than large doses of an antigen, as well mucosal (oral or intranasal) route of administration were more effective in achieving the anti-atherogenic effect. These studies point toward the phenomenon of immune tolerance - a concept we have adopted for development of oral immunomodulators for autoimmune diseases such as AIDS and viral hepatitis B and C. These immune interventions have shown excellent safety profile and high response rates in several clinical trials we have conducted over the last ten years [57,58]. The oral administration of pooled protein fraction derived from adipocytes is likely to induce tolerance to autoantigens involved in lipid metabolism. However, the phenomenon of immune tolerance, which has been discovered more than 100 years ago, still has not been studied well enough to make any authoritative statement in regard to the mechanism of action [16].

## Conclusions

Despite its origin from adipose tissue of pigs, V-6 produces an effect that is opposite to changes in lipid profile resulting from pork fat-based diet [59]. Except anecdotal evidence in alternative diet recipes we are not aware of any credible evidence that eating lard can reduce the risk of heart disease or make us slimmer [60]. On the other hand, animal fat is commonly used in folk medicine for treatment of rheumatism, asthma, and inflammation [61]. The role of inflammation in chronic metabolic disorders such as obesity, type 2 diabetes and CVD is now widely appreciated [1-6]. We are thus at the crossroads between conventional wisdoms and it is clear that further studies are needed to identify the key elements involved in the immune regulation of inflammatory reaction associated with metabolic disorders. The next study will address the immune correlates of V-6 action and seek placebo-controlled confirmation to our preliminary findings in a larger population of patients for an extended period of time.

## Materials and methods

### Subjects

The study involved 9 females and 3 males, all of Asian origin, aged between 22 and 79, with mean/median age 39.8/38 years. The baseline mean body mass index (BMI) was 25.7 kg/m^2^- reflective of higher than normal percentage of body fat - and which places them in overweight category among Asians [1]. Mean waist circumference (WC) in males (97.3 cm) and females (88.7) was above abdominal obesity threshold 90 cm and 80 cm respectively. The baseline HDL cholesterol levels were 39.4 mg/dL which is below 40 mg/dL cut-off normal value. The triglyceride (TG) entry levels were above normal 150 mg/dL, i.e., 168.1 mg/dL. Total cholesterol plasma content was within 200 mg/dL upper limit and LDL content was also within normal range 62-130 mg/dL. Mean systolic and diastolic blood pressure values were also within normal range, i.e., 116.1 and 76.8. Baseline blood glucose content 96.1 mg/dL was normal. Briefly, except normal baseline blood pressure and glucose our patients were overweight or obese and at increased risk of CVD, since they had abnormal baseline BMI, WC, TG, and HDL. Patients consented to receive twice-daily dose of two V-6 pills for two months and be subjected to routine laboratory and physical check-ups at 0.5, 1, and 2 month intervals.

### Lab analyzes

The peripheral blood samples were drawn and sent to a commercial laboratory for complete CBC and standard biochemistry tests including liver, kidney and lipid profile tests.

### Anthropometric measures of adiposity

Mid-arm, abdominal and thigh diameters were measured with a flexible, non-elastic measuring tape at baseline and at 2, 4, and 8 weeks intervals.

### V-6 Immunitor

V-6 is an oral tablet preparation of specially processed pig adipose tissue (fat cells) and is currently approved as a dietary supplement. The tissue was hydrolyzed and protein fraction was precipitated on a magnesium carrier according to proprietary process, which is a modification of earlier published method [16].

### Statistical analysis

Obtained data from study patients analyzed at 2, 4, and 8 week intervals has been analyzed using repeated measure ANOVA test (STATMOST, Dataxiom, Los Angeles, CA). Where appropriate, basic parametric and non-parametric tests were utilized. The probability values for all results were considered significant at p ≤ 0.05.

## Abbreviations

(ALT): Alanine aminotransferase; (TIE2): angiopoietin-2 receptor; (AST): aspartate aminotransferase; (BUN): blood urea nitrogen; (BMI): body mass index; (CVD): cardiovascular disease; (CETP): cholesteryl ester transfer protein; (CBC): complete blood cell; (CI): confidence interval; (CHD): coronary heart disease; (HSP): heat-shock protein; (HDL): high density lipoproteins; (LDL): low density lipoproteins; (TNF-α): tumor necrosis factor alpha; (VEGF): vascular endothelial growth factor receptor 2; (WC): waist circumference.

## Competing interests

Both authors are principal officers and shareholders of the Immunitor company as shown by their affiliation.

## Authors' contributions

ASB performed the statistical analysis and drafted the manuscript. VJ carried out the study. All authors read and approved the final manuscript.
